# Integrating Peptidomics, Network Pharmacology, Molecular Docking, and In Vitro α-Glucosidase Inhibition Assays to Explore the Hypoglycemic Activity and Action Mechanisms of Chinese Yam Peptides

**DOI:** 10.3390/foods15152603

**Published:** 2026-07-25

**Authors:** Hui-Ke Ma, Xin-Yu Li, Liang Shen, Hong-Fang Ji

**Affiliations:** 1 School of Life Sciences, Ludong University, Yantai 264025, China; 2 Institute of Food and Drug Research for One Health, School of Food Engineering, Ludong University, Yantai 264025, China; 3 School of Life Sciences and Medicine, Shandong University of Technology, Zibo 255000, China

**Keywords:** Chinese yam peptides, type 2 diabetes mellitus, peptidomics, network pharmacology, molecular docking

## Abstract

Chinese yam possesses anti-type 2 diabetes mellitus (T2DM) potential. However, existing research has focused on its polysaccharides rather than its abundant peptide components. Therefore, this study integrated peptidomics with network pharmacology and molecular docking to explore the potential anti-T2DM mechanisms of Chinese yam peptides. Firstly, this study revealed that crude peptides extracted from Chinese yam possessed a potential hypoglycemic activity, with an IC_50_ of 568.87 μg/mL against α-glucosidase. Secondly, peptidomics identified 558 peptides with the sequences ranging from 3 to 25 residues. Thirdly, network pharmacology was conducted on 15 candidate peptides selected through bioactivity prediction and safety evaluation. These peptides shared 466 common targets with T2DM, with seven core targets identified through topological network analysis: *SRC*, *EGFR*, *PTPN11*, *PIK3R1*, *ESR1*, *STAT3*, and *PIK3CA*. Enrichment analysis revealed that these targets were significantly associated with PI3K-Akt and MAPK signaling pathways. Fourthly, molecular docking confirmed the strong binding affinities between the 15 bioactive peptides and seven core targets, particularly the high-affinity binding of peptides SIDLYENRL and RAPDDLDTRL to *PIK3CA*. Collectively, these findings demonstrate that Chinese yam peptides possess potential hypoglycemic activity and may ameliorate T2DM through the regulation of insulin signaling via PI3K-Akt activation and the modulation of inflammation via the MAPK pathway.

## 1. Introduction

Diabetes mellitus (DM) is a chronic metabolic disease characterized by persistent hyperglycemia and inadequate or inefficient insulin production. Currently, it has become one of the main public health challenges in the world [1]. Notably, type 2 diabetes mellitus (T2DM) accounts for more than 95% of all cases [2]. The main pathogenetic mechanisms of T2DM are insulin resistance and impairment of insulin secretion owing to the malfunction of pancreatic β-cell [3,4,5]. Furthermore, there are multisystemic complications associated with T2DM, such as non-alcoholic fatty liver disease, and diabetic nephropathy [4,6,7]. According to the epidemiological studies, T2DM also increases the risk for the development of certain malignancies and neurodegenerative diseases [8,9,10], further underlining its systemic effects. Hence, it is clear that T2DM is not only a disease with glucose metabolism disturbance, but a complex, multifactorial disease with widespread organ dysfunction [11].

The prevailing treatment strategy in T2DM includes exogenously injected insulin, as well as oral hypoglycemic drugs, both of which are associated with various limitations [12]. Metformin remains the first-line drug, given its effectiveness in enhancing insulin sensitivity and the extremely favorable cost–benefit ratio [13,14]. More recently, glucagon-like peptide-1 (GLP-1) receptor agonists have attracted much attention as another effective way of treatment for T2DM patients due to their dual benefits in glycemic control and cardiovascular protection [15,16,17]. However, all of these traditional therapies in T2DM treatment have numerous disadvantages that severely limit their long-term therapeutic efficacy. Insulin therapy may induce an undesirable increase in body weight and risk of hypoglycemia [18], while metformin and GLP-1 receptor agonists frequently cause gastrointestinal problems, such as nausea, vomiting, and diarrhea [19,20,21]. All of these adverse effects negatively affect patient compliance and treatment outcomes. Thus, the need to find safer and better-tolerated therapeutic alternatives is obvious, and natural bioactive peptides can become good candidates for antidiabetic drugs.

Naturally derived therapies have gained much prominence as promising treatment alternatives for T2DM due to the benefits of their multi-targeting synergy and favorable safety profiles compared to synthetic pharmaceuticals [22]. Among these, bioactive peptides derived from food have become a hot topic of research in this field due to their natural origin, high safety profile, and well-established therapeutic effects [23,24]. It should be mentioned that the inhibition of α-glucosidase activity is one of the most important ways how food-derived peptides can reduce the level of blood glucose since inhibition of catalytic activity of this enzyme leads to inhibition of carbohydrate hydrolysis and glucose absorption in the intestine, thereby helping to regulate postprandial blood glucose levels [23,25,26]. Although a lot of studies have confirmed the antidiabetic potential of peptides from various food sources [27,28,29], the possibility of employing anti-T2DM activity of many bioactive peptides isolated from Chinese yam has not been explored, and no comprehensive mechanistic studies are available to ascertain their therapeutic efficacy and identify their exact molecular targets in physiological systems.

Network pharmacology has revolutionized the approach to systems biology by providing an all-around analysis of drug–target–disease interactions using multi-omics data in conjunction with advanced bioinformatic tools, which include molecular docking simulations, network topology analysis, and pathway-enrichment algorithms [30,31]. This approach is of particular interest for the study of “medicine–food homology” (MFH) substances since it has proven successful in understanding the inherent multi-component, multi-target therapeutic mechanisms of such compounds. In fact, previous studies have successfully utilized network pharmacology to elucidate the complex anti-T2DM mechanisms of several phytochemicals, thus validating its utility in functional food research [1,32,33]. It should be noted that network pharmacology and molecular docking offer computer simulation-based predictions of mechanisms of action and targets, which require validation in future work.

**Figure 1:**
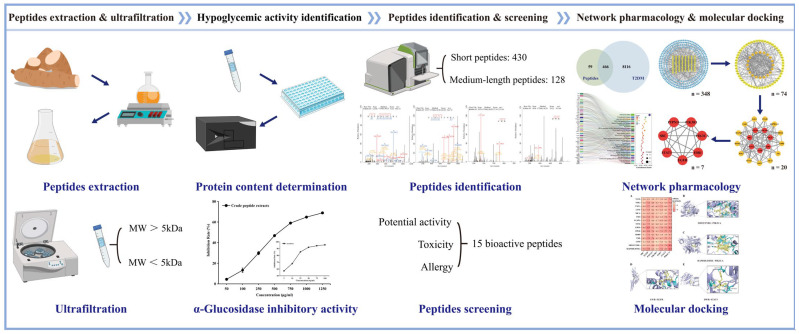
Flowchart of the study design.

According to this well-established method, our study utilized a combination of peptidomics and network pharmacology approaches to systematically investigate the therapeutic potential of bioactive peptides derived from Chinese yam in the management of T2DM (Figure 1).

## 2. Materials and Methods

### 2.1. Materials

Fresh Chinese yam was purchased from a supermarket (Yantai, Shandong Province, China). Cellulase was obtained from Macklin Biochemical Technology Co., Ltd. (Shanghai, China). Neutral protease and 4-nitrophenyl-α-D-glucopyranoside (pNPG) were sourced from Aladdin Biochemical Technology Co., Ltd. (Shanghai, China). Sodium carbonate anhydrous, acarbose, and ready-to-use BCA protein concentration assay kit were purchased from Solarbio Science and Technology Co., Ltd. (Beijing, China). α-Glucosidase was purchased from Yuan Ye Biotechnology Co., Ltd. (Shanghai, China). All enzymes were used and stored according to manufacturers’ specifications.

### 2.2. Enzymatic Digestion of Yam Protein and Peptide Preparation

Bioactive peptides were obtained using the sequential enzymatic hydrolysis method. Fresh Chinese yam was peeled and diced into small pieces before being homogenizing with ultrapure water (1:15, *w*/*v*) in a high-speed homogenizer to prepare a uniform slurry. The hydrolysis process involved two enzymatic stages. (i) Primary hydrolysis: The slurry was adjusted to pH 4.8, and then cellulase was added before incubation at 50 ± 0.5 °C for 100 min under constant agitation. The reaction was stopped by heating the mixture at 100 °C for 10 min in a water bath. (ii) Secondary hydrolysis: The sample was cooled to 25 °C, and then the pH was adjusted to 7.0 with NaOH. Neutral protease was then added in a 6:100 (*w*/*w*) enzyme-to-substrate ratio. The mixture was maintained at 50 ± 0.5 °C for 4 h, with continuous agitation, and then terminated by enzyme inactivation at 100 °C for 10 min. The final hydrolysate was centrifuged at 7600 rpm for 15 min in an Eppendorf 5430 R centrifuge fitted with F-35-6-30 fixed-angle rotor, and the supernatant was collected and stored at −20 °C. For the fractionation of peptides, the supernatant was processed by ultrafiltration using a 5 kDa molecular weight cutoff membrane at 4000 rpm for 8 min. The permeate fraction (<5 kDa) was collected, aliquoted, and stored at −20 °C for bioactivity evaluation and subsequent purification.

### 2.3. Bioactivity Evaluation of Chinese Yam Enzymatic Hydrolysates

#### 2.3.1. Protein-Content Determination

The protein content in Chinese yam enzymatic hydrolysates was determined using a ready-to-use BCA protein concentration assay kit according to the manufacturers’ instructions.

#### 2.3.2. α-Glucosidase Inhibitory Activity Assay

The α-glucosidase inhibitory activity was measured using the pNPG method with some modifications [34]. The experiment included four experimental groups: (i) the sample group (enzyme + sample), (ii) the sample blank group (sample only), (iii) the control group (enzyme only), and (iv) the blank group (buffer only). Procedures involved in the experiment are detailed below: first, 20 μL of α-glucosidase solution (0.2 U/mL) was pre-incubated with 100 μL of sample solutions with different concentrations (50, 100, 250, 500, 750, 1000, and 1250 μg/mL) at 37 °C for 10 min, followed by the addition of 40 μL pNPG substrate (0.25 mM) and further incubation for 20 min. Finally, 50 μL of Na_2_CO_3_ solution (0.2 M) was added to stop the reaction, and the absorbance was measured at 405 nm. The α-glucosidase inhibition rate was calculated according to the following formula:

α-glucosidase inhibition rate (%) = [1 − (A_sample group_ − A_sample blank group_)/(A_control group_ − A_blank group_)] × 100%.

After calculating the inhibition rates at different concentrations, a Logit regression analysis was performed on the data. In preparation for the analysis, concentrations of samples were log10-transformed and used as covariates, while percentage inhibition was taken as the response variable. The IC_50_ value was then interpolated from the fitted regression curve.

### 2.4. Characterization and Screening of Hypoglycemic Peptides

#### 2.4.1. LC-MS/MS-Based Identification of Hypoglycemic Peptides

The <5 kDa peptide fraction was analyzed by LC-MS/MS using a capillary liquid chromatography system coupled to a Q Exactive HF-X mass spectrometer. Chromatographic separation was conducted on a C18 reversed-phase analytical column (150 mm × 0.15 mm, CTInstruments Ltd., Calgary, AB, Canada). Mobile phase consisted of (A) 0.1% (*v*/*v*) formic acid aqueous solution and (B) 0.1% formic acid acetonitrile/aqueous solution (84% acetonitrile), with the following gradient profile: 4–50% B (0–50 min), 50–100% B (50–54 min), and 100% B (54–60 min). Mass spectrometric detection was performed in positive ionization mode, and 10 MS^2^ scans were collected after every full scan. Analysis was carried out using MaxQuant software (v1.5.5.1). The data were searched against the Uniprotkb *Dioscorea* database. The search followed an enzymatic cleavage rule of unspecific and a mass tolerance of 20 ppm for fragment ions and a mass tolerance of 0.1 Da for MS^2^.

#### 2.4.2. Bioactive Peptides Screening and Safety Evaluation

Potential active peptides were identified through systematic analysis using a combined approach. First, peptide bioactivity was predicted using PeptideRanker, with thresholds set at >0.5 probability scores for putative bioactive candidates [35,36]. Next, the peptides were predicted for safety properties with two predictive models: (1) prediction of allergenicity using AlgPred 2.0 (AlgPred 2.0 score < 0.3) [37] and (2) prediction of toxicity using ToxinPred (ToxinPred prediction probability < 0) [38]. Only peptides satisfying all three criteria (bioactivity score > 0.5, non-allergenic prediction, and non-toxic classification) were selected for further experiments.

### 2.5. Target Prediction for Chinese Yam Bioactive Peptides

For comprehensive target prediction, the 2D structures of 15 bioactive peptides were analyzed using PharmMapper (Z’-score cutoff ≥ 0.5) [32] and SwissTargetPrediction databases [39]. In SwissTargetPrediction database, the organism option was set to *Homo sapiens*. Targets that appeared in databases were merged, eliminating any duplicates based on the UniProt ID, thus ensuring target specificity for subsequent analyses.

### 2.6. Identification of T2DM-Associated Therapeutic Targets

In order to make sure that the research is complete, a thorough retrieval strategy utilizing standardized medical subject headings, including but not limited to “T2DM”, “type 2 diabetes mellitus”, and “Diabetes Mellitus, Type 2”, was used. This study performed a comprehensive search for potential T2DM-related targets in three major databases: Therapeutic Target Database (TTD), GeneCards (with a relevance score threshold > 10), and OMIM. The targets retrieved from the abovementioned databases through the use of the above search criteria were compiled, and any possible duplication of the target was eliminated to ensure its uniqueness.

### 2.7. Construction and Analysis of Protein–Protein Interaction Network

Potential targets for the intervention of T2DM by Chinese yam peptides were determined using jvenn with cross-tabulation of peptide-predicted targets and T2DM disease-related targets, and the results were presented via Venn diagrams. These overlapping targets were further used to generate a protein–protein interaction (PPI) network through STRING database with the following parameters: species set to *Homo sapiens*, minimum required interaction score of 0.9, and exclusion of disconnected nodes [40]. The resulting network file was exported in TSV format and imported to Cytoscape (v3.10.0) for topological analysis using CytoNCA 2.1.6 plugin [41]. Six centrality metrics were calculated for each node: Betweenness Centrality (BC), Closeness Centrality (CC), Degree Centrality (DC), Eigenvector Centrality (EC), Local Average Connectivity-based method (LAC), and Network Centrality (NC). Core targets were identified in an iterative process in which only those nodes with scores above the median for all six parameters were retained [42].

### 2.8. Functional Enrichment and Network Analysis

In order to elucidate the mechanistic basis of yam peptides’ anti-T2DM activity, we carried out a thorough functional annotation of the 74 cross-targets using Metascape with the following analytical pipeline: Gene Ontology (GO) enrichment covering the categories of biological processes (BPs), molecular functions (MFs), and cellular components (CCs), and pathway analysis using the Kyoto Encyclopedia of Genes and Genomes (KEGG) database [43]. All analyses were strictly done for “*Homo sapiens*”, and the enrichment results were processed using *p* < 0.05 as the screening criterion. For systems-level interpretation, we established an integrated peptide–target–pathway–disease network in Cytoscape (v3.10.0), revealing the complex therapeutic mechanism of Chinese yam peptide intervention in T2DM.

### 2.9. Molecular Docking Analysis

The interaction between 7 prioritized core targets and 15 bioactive peptides extracted from Chinese yam was investigated using molecular docking analysis. The 2D structures of peptides were generated and subsequently converted to optimized 3D conformations with energy minimization. Target protein structures were obtained from the PDB database, while water molecules and original ligands were removed using PyMOL 2.4.0a0 software [44], followed by hydrogen addition and structure optimization. Blind docking of processed core target proteins as receptors and bioactive peptides as ligands was carried out using AutoDock Vina 1.1.2 [45].

## 3. Results

### 3.1. Protein-Content Determination and α-Glucosidase Inhibitory Activity Analysis

**Figure 2:**
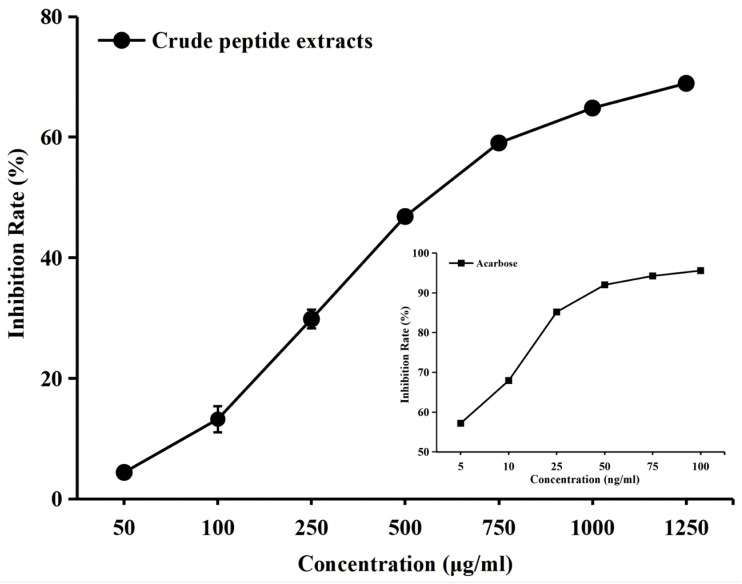
α-Glucosidase inhibitory activity analysis of the crude peptide extracts from Chinese yam.

The quantification of protein concentration of Chinese yam enzymatic hydrolysates using the ready-to-use BCA protein concentration assay kit revealed a concentration of 1285 μg/mL, indicating efficient protein hydrolysis in the enzymatic hydrolysis procedure. The α-glucosidase inhibition activity of the sample was evaluated at seven concentration levels (50–1250 μg/mL) and exhibited dose-dependent inhibition (Figure 2). Notably, the maximum inhibition rate of the crude peptide extracts reached 68.92% at the highest tested concentration (1250 μg/mL), indicating its α-glucosidase inhibitory potential. The IC_50_ of the enzymatic hydrolysates was determined to be 568.87 μg/mL, higher than that of the reference drug acarbose (IC_50_ = 3.979 ng/mL).

### 3.2. LC-MS/MS Characterization of Yam-Derived Peptides

**Figure 3:**
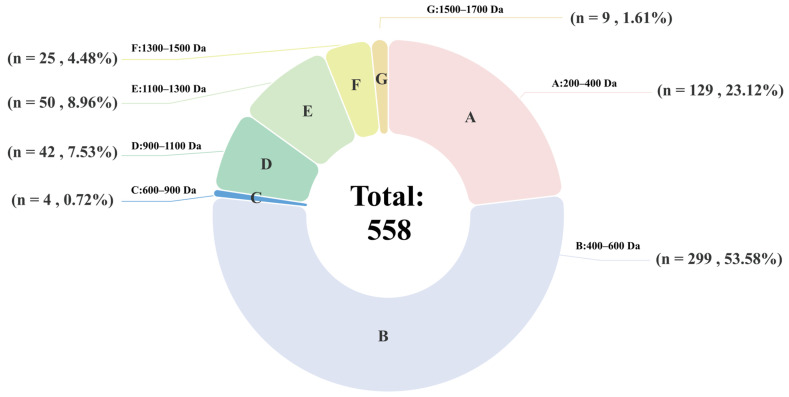
Molecular weight distribution profile of Chinese yam hydrolysate peptides.

In total, 558 peptide sequences were identified using the high-resolution mass spectrometric analysis in the hydrolysates of Chinese yam, as shown in Figure 3. These peptides were classified according to chain length distribution. The peptide distribution consisted of 430 oligopeptides (77.1% of the total), which ranged from 3 to 7 amino acid residues, while the other 128 peptides (22.9%) contained 8–25 residues. Notably, 98.4% (n = 549) of all detected peptides had molecular weights below 1500 Da, a characteristic molecular weight range for bioactive peptides.

### 3.3. Bioinformatics Screening and Safety Profiling of Bioactive Peptides

**Figure 4:**
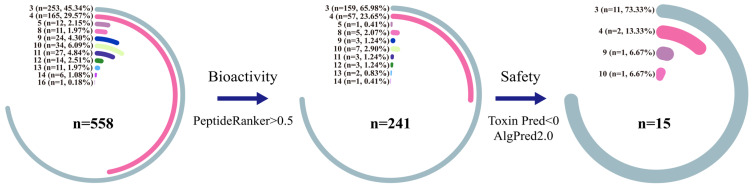
Bioinformatics screening and safety profiling of bioactive peptides.

To identify novel candidates of anti-T2DM peptide, a systematic bioinformatics screening pipeline was developed based on 558 identified yam peptides (Figure 4) [46]. When PeptideRanker was used to predict the bioactivity of these peptides, 241 peptides (43.2% of total) were predicted to have bioactivity, with scores ranging from 0.5016 to 0.9973. Subsequent safety evaluation eliminated peptides exhibiting potential allergenicity or toxicity. As a result of this rigorous selection process, 15 high-confidence peptide candidates (2.7% of initial pool) were selected (Table 1).

### 3.4. Target Prediction Analysis of Bioactive Peptides

Comprehensive target profiling of the 15 selected yam peptides was performed by employing complementary prediction approaches. The screening process via the SwissTargetPrediction method generated 856 potential human protein targets, while PharmMapper analysis yielded 1668 putative targets. After implementing stringent quality controls, 525 valid targets were finally obtained.

### 3.5. Systematic Identification of T2DM-Associated Therapeutic Targets

A comprehensive multi-database mining approach was employed to identify T2DM-related targets with the use of search terms (“T2DM”; “type 2 diabetes”; and “Diabetes Mellitus, Type 2”), yielding 8582 non-redundant targets associated with T2DM pathogenesis. The initial search obtained 8487 targets from GeneCards, 99 targets from TTD, and 272 targets from OMIM.

### 3.6. PPI Network Analysis of Common Targets Between Chinese Yam Peptides and T2DM

**Figure 5:**
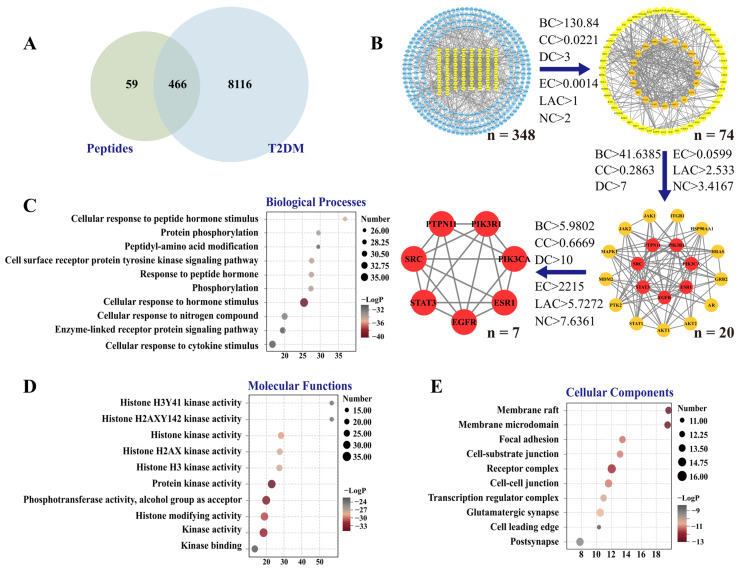
Network pharmacology analysis of Chinese yam bioactive peptides and T2DM. (**A**) Intersection target Venn diagram. Venn diagram showing 466 overlapping targets between the 525 predicted peptide targets (green) and 8582 T2DM-related targets (blue) identified through database mining. (**B**) Screening workflow and criteria for core target identification in the Chinese yam peptide–T2DM interaction study. (**C**–**E**) GO enrichment analysis of potential genes for Chinese yam bioactive peptides anti-T2DM.

After performing the intersection analysis on the 525 yam peptide targets and the 8582 T2DM-related targets, 466 overlapping targets were found (Figure 5A). After importing the common targets into the STRING database, a PPI network with high-confidence interactions was generated, yielding a network of 348 nodes and 875 edges after removing unconnected proteins. Using the CytoNCA 2.1.6 plugin of Cytoscape (v3.10.0), we calculated BC, CC, DC, EC, LAC, and NC of the targets. Through three rounds of screening by taking the median value of these parameters (Figure 5B), we identified seven core targets—*SRC*, *EGFR*, *PTPN11*, *PIK3R1*, *ESR1*, *STAT3*, and *PIK3CA*—that exhibited the highest network centrality and were selected for further mechanistic investigation.

### 3.7. Functional Enrichment Analysis of Core Targets

**Figure 6:**
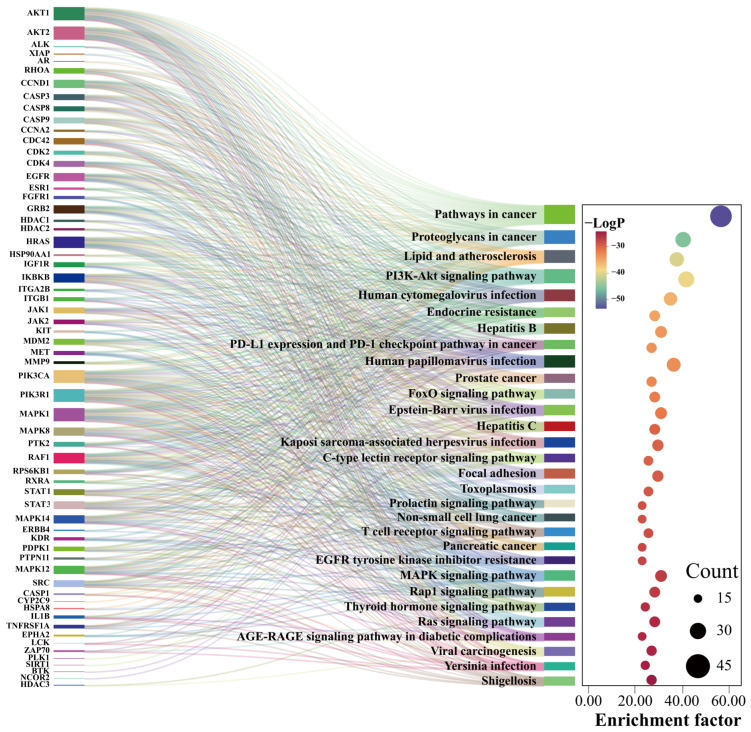
KEGG enrichment analysis of potential genes for Chinese yam bioactive peptides anti-T2DM.

Functional annotation of the 74 targets through GO enrichment analysis revealed significant associations across all three ontological categories. In the BP domain, the targets were predominantly enriched in response to stimulus, positive regulation of biological processes, and cellular metabolic processes (Figure 5C). For MF, important enrichments were shown in the following: kinase activity and histone-modifying activity (Figure 5D). CC analysis highlighted membrane raft, membrane microdomain, and receptor complex (Figure 5E). KEGG pathway enrichment (*p* < 0.05, Figure 6) further revealed that these targets were significantly enriched in T2DM-relevant pathways, including lipid and atherosclerosis, PI3K-Akt signaling pathway, EGFR tyrosine kinase inhibitor resistance, MAPK signaling pathway, and AGE-RAGE signaling pathway in diabetic complications. These findings suggest that the peptides from Chinese yam might ameliorate T2DM via multi-pathway regulation, particularly by modulating insulin signaling (PI3K-Akt), inflammatory responses (AGE-RAGE and MAPK), and metabolic dysfunction (lipid metabolism).

### 3.8. Molecular Docking of Yam Peptide–Target Interactions

**Figure 7:**
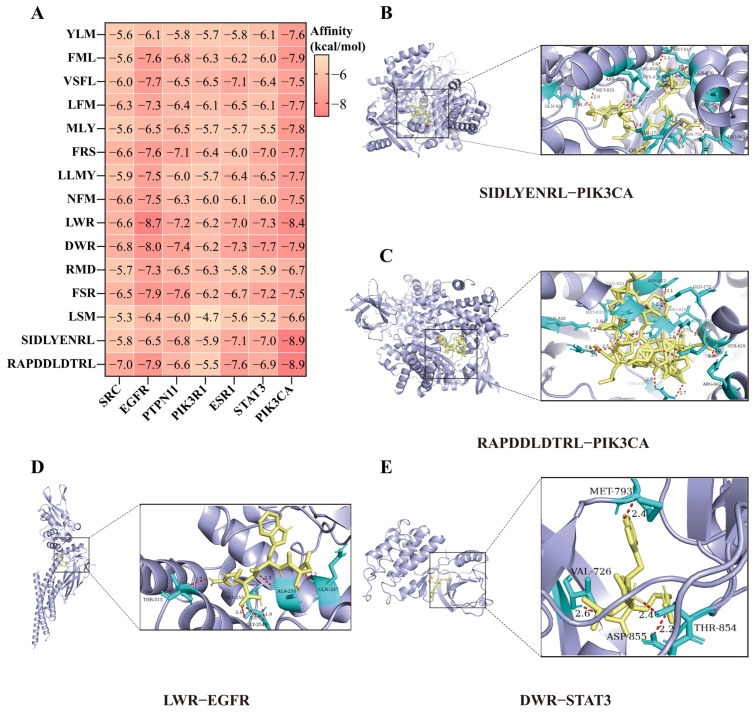
The binding affinity and modes of 15 bioactive peptides from Chinese yam with 7 core targets through molecular docking. (**A**) The binding affinity of Chinese yam peptides (vertical axis) and core target proteins (horizontal axis), with color intensity corresponding to docking scores (kcal/mol) ranging from −4.7 (weak, yellow) to −8.9 (strong, red). (**B**) The binding mode of SIDLYENRL and Phosphatidylinositol-4,5-Bisphosphate 3-Kinase Catalytic Subunit Alpha (*PIK3CA*). (**C**) The binding mode of RAPDDLDTRL and *PIK3CA*. (**D**) The binding mode of LWR and Epidermal Growth Factor Receptor (*EGFR*). (E) The binding mode of DWR and Signal Transducer and Activator of Transcription 3 (*STAT3*). In panels (**B**–**E**), the red dashed lines represent hydrogen bonds formed between the peptide and amino acid residues of the target protein, with the corresponding bond lengths labeled.

The binding interactions between 15 bioactive peptides extracted from yam and seven core target proteins that have been identified by network analysis were investigated by molecular docking simulations. As shown in the binding energy heatmap (Figure 7A), two peptides (SIDLYENRL and RAPDDLDTRL) (Figure 7B,C) showed particularly strong binding affinity towards the *PIK3CA*, with the same docking score of −8.9 kcal/mol. Likewise, the peptide LWR exhibited considerable interaction with *EGFR* (−8.7 kcal/mol) (Figure 7D), while the peptide DWR displayed significant interaction with *STAT3* (−7.7 kcal/mol) (Figure 7E).

From the analysis of the binding energy, it was found that the combination of *PIK3CA*, *EGFR*, and *STAT3* had the most favorable binding energies when compared with other target proteins investigated, implying that these three targets might play an important role in contributing to the hypoglycemic activity mediated by yam peptides. It has also been indicated from the mode of molecular interaction that both SIDLYENRL and RAPDDLDTRL established multiple hydrogen bonds with key residues present in the kinase domain of *PIK3CA*. These findings have provided structural evidence for the possible pharmacological potential of yam peptides to target the key signaling pathways associated with T2DM, especially targeting key regulators of insulin signaling (*PIK3CA*), cellular proliferation (*EGFR*), and inflammatory responses (*STAT3*). Strong binding affinities of these peptide–target pairs merit further experimental validation to confirm their therapeutic potential.

## 4. Discussion

T2DM is a multifactorial metabolic disorder [47,48] whose ever-increasing global prevalence and complications remain one of the major challenges to health systems worldwide, thus creating an urgent need for developing safer therapeutic options compared to conventional hypoglycemic agents [49]. Chinese yam, a well-characterized MFH substance [50,51], has been recognized as a promising source of bioactive compounds with antidiabetic potentials [52,53,54,55]. Although many studies have clarified the glucose-lowering mechanisms of yam polysaccharides [56,57,58,59], the hypoglycemic potential of yam peptides has not yet been studied extensively. This study, by integrating peptidomics and network pharmacology with a molecular docking approach, firstly demonstrated that peptides derived from Chinese yam exert α-glucosidase inhibitory activity and systematically investigated the possible mechanisms by which Chinese yam peptides exert their hypoglycemic effects, thus filling a very critical gap in research.

Since the inhibition of α-glucosidase is one of the methods that help to regulate postprandial hyperglycemia in T2DM management [27], we investigated the α-glucosidase inhibitory activity of the enzymatic hydrolysates of yam peptides. It was found that while the α-glucosidase inhibitory activity of the enzymatic hydrolysates (IC_50_ = 568.87 μg/mL) was lower than that of the positive control acarbose (IC_50_ = 3.979 ng/mL), the active peptides derived from yam exhibited superior biosafety and lower risks of adverse effects [60]. Moreover, compared with α-glucosidase inhibitors reported in other works from the literature, the IC_50_ value of Chinese yam peptides in this study was relatively low, indicating that these peptides have better hypoglycemic activity and wider applications in T2DM treatment [27,61,62]. Considering the variety of peptides in the crude extract, the antidiabetic effect of these peptides may be mediated by the regulation of a number of targets and signaling pathways. Accordingly, in light of the above in vitro activity data, we employed network pharmacology methods to predict the potential targets and signaling pathways for the selected peptides in the Chinese yam peptides.

Subsequently, 15 high-priority peptides were identified which not only showed potential hypoglycemic activity but were also considered safe based on LC-MS/MS analysis and systematic computational screening. In the amino acid composition analysis of these peptides, Arg, Glu, and Leu residues were found to be highly abundant. These residues are the characteristics of α-glucosidase inhibitory peptides, as mentioned before. They may enhance the binding affinity of peptides towards α-glucosidase by facilitating intermolecular interactions, thus synergistically promoting their inhibitory activity [62]. In addition, 525 non-redundant protein targets of peptides were obtained using two complementary approaches. Meanwhile, systematic searches in disease databases have provided 8582 T2DM-associated targets. This robust dataset became the foundation for further network pharmacology analysis. Through an overlap analysis of the aforementioned datasets, a total of 466 common targets were identified. This integrated approach serves as the basis for understanding how yam peptides can modulate T2DM pathophysiology at a molecular level, and their food-grade origin suggests great translational potential as nutraceuticals.

PPI network analysis was performed on the overlapping targets of Chinese yam bioactive peptides and T2DM-associated proteins and identified seven core target genes: *SRC*, *EGFR*, *PTPN11*, *PIK3R1*, *ESR1*, *STAT3*, and *PIK3CA*. Extensive evidence implicates these core targets in the pathogenesis of T2DM by three principal mechanisms. (i) Core regulatory role of the PI3K-Akt signaling axis: As important members of the phosphatidylinositol 3-kinase (PI3K) family, *PIK3R1* and *PIK3CA* are involved in the core PI3K-Akt signaling pathway and exert essential regulation on glucose metabolism [63]. *EGFR*, an upstream factor of the PI3K-Akt pathway, has a direct impact on the activation of the PI3K-Akt pathway [64,65]. *SRC* tyrosine kinase regulates various biological processes through the phosphorylation of tyrosine residues [66], including modulation of both PI3K-Akt signaling and lipid metabolism [67]. The phosphorylation of *STAT3* upregulates suppressor of cytokine signaling 3 expression, resulting in inhibition of PI3K-Akt phosphorylation and impairment of insulin sensitivity by modulating glucose homeostasis through the action on PI3K-Akt signaling [68]. (ii) Molecular basis of insulin resistance. In insulin signaling transduction, *PTPN11*, a protein tyrosine phosphatase family member, impedes the activity of the insulin receptor and its substrates and plays a crucial role in the development of insulin resistance [69]. (iii) Metabolic regulation of inflammatory responses. Furthermore, the expression levels of *ESR1* are significantly related to the inflammatory response. *ESR1* primarily encodes the estrogen receptor, and in the case of estrogen deficiency, there is an excessive production of pro-inflammatory cytokines (such as TNF-α and IL-6), thereby worsening insulin resistance [70,71].

Functional enrichment analysis was performed for the 74 candidate targets using GO and KEGG databases. In KEGG pathway analysis, we found significant enrichment in five key pathways: lipid and atherosclerosis, PI3K-Akt signaling pathway, EGFR tyrosine kinase inhibitor resistance, MAPK signaling pathway, and the AGE-RAGE signaling pathway in diabetic complications. The PI3K/Akt signaling pathway, a well-characterized regulator in T2DM pathogenesis, coordinates glucose homeostasis through three distinct mechanisms [72]. First, it facilitates the translocation of glucose transporter type 4 vesicles from intracellular compartments to the plasma membrane by Akt-mediated phosphorylation of TBC1 domain family member 4, thus enhancing peripheral glucose uptake [73,74,75]. Second, the phosphorylation-induced inactivation of glycogen synthase kinase 3 beta triggers glycogen synthase activity, promoting hepatic glycogen storage [76]. Third, the Akt-mediated phosphorylation of forkhead box O1 transcription factors decreases the transcription of the hepatic gluconeogenic genes encoding rate-limiting enzymes glucose-6-phosphatase and phosphoenolpyruvate carboxykinase, which suppresses hepatic gluconeogenesis [76,77]. The MAPK pathway mediates the development of T2DM through its three major subfamilies: p38 mitogen-activated protein kinase (p38 MAPK), c-Jun N-terminal kinase, and extracellular signal-regulated kinase 1/2 [71,78]. Of particular importance is the fact that p38 MAPK activation leads to transcription of proinflammatory cytokines such as TNF-α and IL-6 via NF-κB, collectively establishing a chronic inflammatory state that sustains insulin resistance [79]. Experimental evidence has shown that the inhibition of p38 MAPK not only enhances insulin sensitivity but also promotes glucose transport in adipocytes [79].

Molecular docking was conducted between the 15 yam peptides and seven core targets to investigate the structural interactions between yam peptides and their targets. The analysis of molecular docking demonstrated significant binding affinities between the 15 bioactive yam peptides and three core targets, namely *PIK3CA* (−8.9 to −6.6 kcal/mol), *EGFR* (−8.7 to −6.1 kcal/mol), and *STAT3* (−7.7 to −5.2 kcal/mol), thus indicating favorable molecular interactions. Among these, the peptides SIDLYENRL and RAPDDLDTRL showed high affinities for *PIK3CA* (ΔG = −8.9 kcal/mol), while the peptide LWR exhibited strong binding to *EGFR* (ΔG = −8.7 kcal/mol).

In particular, integrating the results obtained from molecular docking with KEGG pathway analysis helped us to suggest that bioactive yam peptides might exert anti-T2DM effects through a multi-target mechanism of action involving parallel modulation of metabolic and inflammatory pathways. The high binding affinity of some peptides, such as SIDLYENRL and RAPDDLDTRL, to *PIK3CA*, which encodes the catalytic subunit of PI3K, indicates a direct promotion of insulin signaling and glucose uptake along the PI3K-Akt axis. Simultaneously, the binding of peptides to *EGFR* and *STAT3* could serve as an additional way to regulate the inflammation and insulin sensitivity via the MAPK pathway. Thus, the two-pathway intervention strategy might address metabolic defects via PI3K-Akt, while the inflammatory component is targeted via the MAPK/STAT3 in the pathogenesis of T2DM, hence representing a comprehensive therapeutic approach that typically characterizes traditional MFH substances. However, it must be stated that the above analysis of peptide interactions with receptors and the signaling pathways involved was carried out using network pharmacology and molecular docking simulations, and hence, needs to be validated through a number of experiments.

In this study, peptidomics, in vitro experiments, network pharmacology, and molecular docking were combined to develop a highly efficient and economically favorable screening strategy. Furthermore, 15 potential hypoglycemic candidate peptides were screened out, and the mechanisms of action of these peptides were proposed based on the results from computational analysis. However, there are still some limitations in this study. Experimentally, the determination of α-glucosidase inhibitory activity was carried out using a crude extract of Chinese yam with a molecular weight of <5 kDa, rather than the 15 individual candidate peptides. The inhibition activity shown by this extract could be the result of the cooperation between one, several, or all of these peptides; what contribution does each peptide make and how much is still unknown and needs to be proved by subsequent experiment. Computationally, AutoDock Vina might yield less-than-accurate docking conformations of decapeptides, necessitating further validation through additional experiments.

## 5. Conclusions

This study, based on both in vitro experiment and computational prediction, has revealed the α-glucosidase inhibitory activity of a low-molecular-weight peptide fraction (<5 kDa) derived from Chinese yam and systematically predicted its possible multi-target mechanisms. Experimental results proved that the low-molecular-weight peptide fraction derived from Chinese yam exhibited α-glucosidase inhibitory activity. As a result, according to this finding, 15 peptides derived from the low-molecular-weight peptide fraction are predicted to act synergistically by regulating the PI3K-Akt and MAPK/STAT3 signaling pathways at once through targeting key nodes such as *PIK3CA*, *EGFR*, and *STAT3*. The multi-target mechanism predicted here includes enhancement of insulin sensitivity through PI3K-Akt activation in combination with the reduction of inflammatory responses via regulation of the MAPK/STAT3 pathway, which effectively deals with two fundamental pathologies in T2DM. In addition to providing molecular insights into the potential therapeutic mechanisms of traditional MFH compounds, this strategy can be used as a major resource for developing novel functional ingredients targeting T2DM. This comprehensive analysis of the complex multi-target mechanism of bioactive peptides bridges traditional knowledge with modern pharmacological research. However, the conclusions presented above need to be verified through further experiments.

## Figures and Tables

**Table 1:** Characterization of yam-derived bioactive peptides identified through integrated bioinformatics screening.

Number	Single-Letter Amino Acid Code	PeptideRanker Score	Toxicity	Allergy
1	YLM	0.8021	Non-toxin	Non-allergen
2	FML	0.9851	Non-toxin	Non-allergen
3	VSFL	0.5803	Non-toxin	Non-allergen
4	LFM	0.9803	Non-toxin	Non-allergen
5	MLY	0.7818	Non-toxin	Non-allergen
6	FRS	0.8204	Non-toxin	Non-allergen
7	LLMY	0.6803	Non-toxin	Non-allergen
8	NFM	0.9601	Non-toxin	Non-allergen
9	LWR	0.9375	Non-toxin	Non-allergen
10	DWR	0.8769	Non-toxin	Non-allergen
11	RMD	0.5056	Non-toxin	Non-allergen
12	FSR	0.8312	Non-toxin	Non-allergen
13	LSM	0.5137	Non-toxin	Non-allergen
14	SIDLYENRL	0.5207	Non-toxin	Non-allergen
15	RAPDDLDTRL	0.6279	Non-toxin	Non-allergen

## Data Availability

The raw data supporting the conclusions of this article will be made available by the authors on request.

## References

[1] Ononamadu C.J., Seidel V. (2024). Exploring the Antidiabetic Potential of Salvia officinalis Using Network Pharmacology, Molecular Docking and ADME/Drug-Likeness Predictions. Plants.

[2] Zhang L., Chu J., Hao W., Zhang J., Li H., Yang C., Yang J., Chen X., Wang H. (2021). Gut Microbiota and Type 2 Diabetes Mellitus: Association, Mechanism, and Translational Applications. Mediat. Inflamm..

[3] DeFronzo R.A., Ferrannini E., Groop L., Henry R.R., Herman W.H., Holst J.J., Hu F.B., Kahn C.R., Raz I., Shulman G.I. (2015). Type 2 diabetes mellitus. Nat. Rev. Dis. Prim..

[4] Zhou Z., Sun B., Yu D., Zhu C. (2022). Gut Microbiota: An Important Player in Type 2 Diabetes Mellitus. Front. Cell. Infect. Microbiol..

[5] Galicia-Garcia U., Benito-Vicente A., Jebari S., Larrea-Sebal A., Siddiqi H., Uribe K.B., Ostolaza H., Martín C. (2020). Pathophysiology of Type 2 Diabetes Mellitus. Int. J. Mol. Sci..

[6] Younossi Z.M., Golabi P., de Avila L., Paik J.M., Srishord M., Fukui N., Qiu Y., Burns L., Afendy A., Nader F. (2019). The global epidemiology of NAFLD and NASH in patients with type 2 diabetes: A systematic review and meta-analysis. J. Hepatol..

[7] Tilg H., Moschen A.R., Roden M. (2017). NAFLD and diabetes mellitus. Nat. Rev. Gastroenterol. Hepatol..

[8] Sabari S.S., Balasubramani K., Iyer M., Sureshbabu H.W., Venkatesan D., Gopalakrishnan A.V., Narayanaswamy A., Senthil Kumar N., Vellingiri B. (2023). Type 2 Diabetes (T2DM) and Parkinson’s Disease (PD): A Mechanistic Approach. Mol. Neurobiol..

[9] Zhang Y.Y., Li Y.J., Xue C.D., Li S., Gao Z.N., Qin K.R. (2024). Effects of T2DM on cancer progression: Pivotal precipitating factors and underlying mechanisms. Front. Endocrinol..

[10] Cosmin Stan M., Paul D. (2024). Diabetes and Cancer: A Twisted Bond. Oncol. Rev..

[11] Tao T., Deng P., Wang Y., Zhang X., Guo Y., Chen W., Qin J. (2022). Microengineered Multi-Organoid System from hiPSCs to Recapitulate Human Liver-Islet Axis in Normal and Type 2 Diabetes. Adv. Sci..

[12] Drake T., Landsteiner A., Langsetmo L., MacDonald R., Anthony M., Kalinowski C., Ullman K., Billington C.J., Kaka A., Sultan S. (2024). Newer Pharmacologic Treatments in Adults With Type 2 Diabetes: A Systematic Review and Network Meta-analysis for the American College of Physicians. Ann. Intern. Med..

[13] Foretz M., Guigas B., Viollet B. (2023). Metformin: Update on mechanisms of action and repurposing potential. Nat. Rev. Endocrinol..

[14] LaMoia T.E., Shulman G.I. (2021). Cellular and Molecular Mechanisms of Metformin Action. Endocr. Rev..

[15] Vilsbøll T., Christensen M., Junker A.E., Knop F.K., Gluud L.L. (2012). Effects of glucagon-like peptide-1 receptor agonists on weight loss: Systematic review and meta-analyses of randomised controlled trials. BMJ.

[16] Yao H., Zhang A., Li D., Wu Y., Wang C.Z., Wan J.Y., Yuan C.S. (2024). Comparative effectiveness of GLP-1 receptor agonists on glycaemic control, body weight, and lipid profile for type 2 diabetes: Systematic review and network meta-analysis. BMJ.

[17] Simms-Williams N., Treves N., Yin H., Lu S., Yu O., Pradhan R., Renoux C., Suissa S., Azoulay L. (2024). Effect of combination treatment with glucagon-like peptide-1 receptor agonists and sodium-glucose cotransporter-2 inhibitors on incidence of cardiovascular and serious renal events: Population based cohort study. BMJ.

[18] Galdón Sanz-Pastor A., Justel Enríquez A., Sánchez Bao A., Ampudia-Blasco F.J. (2024). Current barriers to initiating insulin therapy in individuals with type 2 diabetes. Front. Endocrinol..

[19] Weinberg Sibony R., Segev O., Dor S., Raz I. (2023). Drug Therapies for Diabetes. Int. J. Mol. Sci..

[20] Bonnet F., Scheen A. (2017). Understanding and overcoming metformin gastrointestinal intolerance. Diabetes Obes. Metab..

[21] Sun F., Yu K., Yang Z., Wu S., Zhang Y., Shi L., Ji L., Zhan S. (2012). Impact of GLP-1 receptor agonists on major gastrointestinal disorders for type 2 diabetes mellitus: A mixed treatment comparison meta-analysis. Exp. Diabetes Res..

[22] Fan S., Liu Q., Du Q., Zeng X., Wu Z., Pan D., Tu M. (2024). Multiple roles of food-derived bioactive peptides in the management of T2DM and commercial solutions: A review. Int. J. Biol. Macromol..

[23] Yu J., Chen G., Jin Y., Zhang M., Wu T. (2025). Research Progress of Bioactive Peptides in Improving Type II Diabetes. Foods.

[24] Antony P., Vijayan R. (2021). Bioactive Peptides as Potential Nutraceuticals for Diabetes Therapy: A Comprehensive Review. Int. J. Mol. Sci..

[25] Jahandideh F., Bourque S.L., Wu J. (2022). A comprehensive review on the glucoregulatory properties of food-derived bioactive peptides. Food Chem. X.

[26] Rahmi A., Arcot J. (2023). In Vitro Assessment Methods for Antidiabetic Peptides from Legumes: A Review. Foods.

[27] Wang X., Deng Y., Xie P., Liu L., Zhang C., Cheng J., Zhang Y., Liu Y., Huang L., Jiang J. (2023). Novel bioactive peptides from ginkgo biloba seed protein and evaluation of their α-glucosidase inhibition activity. Food Chem..

[28] Sun D., Zheng X., Zhang M., Pan J., Lu Y. (2026). High-Value Utilization of Coconut Kernel Fiber By-Products: The Insulin-Sensitizing Effect of Novel α-Glucosidase-Inhibiting Peptides Derived from Coconut Kernel Fiber on T2DM Mice. Foods.

[29] Zhu Y., Zhou E., Tang Y., Li Q., Wu L. (2025). Edible Yellow Mealworm-Derived Antidiabetic Peptides: Dual Modulation of α-Glucosidase and Dipeptidyl-Peptidase IV Inhibition Revealed by Integrated Proteomics, Bioassays, and Molecular Docking Analysis. Foods.

[30] Zhao L., Zhang H., Li N., Chen J., Xu H., Wang Y., Liang Q. (2023). Network pharmacology, a promising approach to reveal the pharmacology mechanism of Chinese medicine formula. J. Ethnopharmacol..

[31] Li X., Liu Z., Liao J., Chen Q., Lu X., Fan X. (2023). Network pharmacology approaches for research of Traditional Chinese Medicines. Chin. J. Nat. Med..

[32] Liu T., Zhu C., Duan Z., Ma P., Ma X., Fan D. (2024). Network Pharmacological Analysis Combined with Experimental Verification to Explore the Effect of Ginseng Polypeptide on the Improvement of Diabetes Symptoms in db/db Mice. J. Agric. Food Chem..

[33] Fatimawali, Tallei T.E., Kepel B.J., Bodhi W., Manampiring A.E., Nainu F. (2023). Molecular Insight into the Pharmacological Potential of *Clerodendrum minahassae* Leaf Extract for Type-2 Diabetes Management Using the Network Pharmacology Approach. Medicina.

[34] Li N., Liu K., Zhang Y., Hui Z., Wang P., Sun S., Du C. (2025). Identification of novel α-glucosidase inhibitory peptides in Meretrix meretrix Linnaeus and their inhibitory kinetics using in silico and in vitro analyses. Int. J. Biol. Macromol..

[35] Gao Y.F., Liu M.Q., Li Z.H., Zhang H.L., Hao J.Q., Liu B.H., Li X.Y., Yin Y.Q., Wang X.H., Zhou Q. (2023). Purification and identification of xanthine oxidase inhibitory peptides from enzymatic hydrolysate of α-lactalbumin and bovine colostrum casein. Food Res. Int..

[36] Quan Z., Wang Z., Wang Z., Hou Z., Liu B., Guo X., Zhu B., Hu Y. (2025). Study on the antioxidant and antiosteoporotic activities of the oyster peptides prepared by ultrasound-assisted enzymatic hydrolysis. Ultrason. Sonochem..

[37] Sharma N., Patiyal S., Dhall A., Pande A., Arora C., Raghava G.P.S. (2021). AlgPred 2.0: An improved method for predicting allergenic proteins and mapping of IgE epitopes. Brief. Bioinform..

[38] Gupta S., Kapoor P., Chaudhary K., Gautam A., Kumar R., Raghava G.P.S. (2015). Peptide toxicity prediction. Methods Mol. Biol..

[39] Daina A., Michielin O., Zoete V. (2019). SwissTargetPrediction: Updated data and new features for efficient prediction of protein targets of small molecules. Nucleic Acids Res..

[40] Szklarczyk D., Kirsch R., Koutrouli M., Nastou K., Mehryary F., Hachilif R., Gable A.L., Fang T., Doncheva N.T., Pyysalo S. (2023). The STRING database in 2023: Protein-protein association networks and functional enrichment analyses for any sequenced genome of interest. Nucleic Acids Res..

[41] Shannon P., Markiel A., Ozier O., Baliga N.S., Wang J.T., Ramage D., Amin N., Schwikowski B., Ideker T. (2003). Cytoscape: A software environment for integrated models of biomolecular interaction networks. Genome Res..

[42] Li X.-Y., Shen L., Ji H.-F. (2025). Exploring the anti-Alzheimer’s disease mechanisms of edible medicinal plant Polygonum cuspidatum by integrating network pharmacology and molecular docking. Ind. Crops Prod..

[43] Zhou Y., Zhou B., Pache L., Chang M., Khodabakhshi A.H., Tanaseichuk O., Benner C., Chanda S.K. (2019). Metascape provides a biologist-oriented resource for the analysis of systems-level datasets. Nat. Commun..

[44] Delano W.L. (2002). The PyMol Molecular Graphics System. Proteins Struct. Funct. Bioinform..

[45] Trott O., Olson A.J. (2010). AutoDock Vina: Improving the speed and accuracy of docking with a new scoring function, efficient optimization, and multithreading. J. Comput. Chem..

[46] Ouyang Y., Yue Y., Wu N., Wang J., Geng L., Zhang Q. (2024). Identification and anticoagulant mechanisms of novel factor XIa inhibitory peptides by virtual screening of a in silico generated deep-sea peptide database. Food Res. Int..

[47] Chen L., Wang J., Ren Y., Ma Y., Liu J., Jiang H., Liu C. (2024). Artesunate improves glucose and lipid metabolism in db/db mice by regulating the metabolic profile and the MAPK/PI3K/Akt signalling pathway. Phytomedicine.

[48] Hu Z., Yang M., Yang L., Xie C., Gao H., Fu X., Xie H., Liu Y. (2020). Network Pharmacology-Based Identification of the Mechanisms of Shen-Qi Compound Formula in Treating Diabetes Mellitus. Evid.-Based Complement. Altern. Med..

[49] Desai V., Shaikhsurab M.Z., Varghese N., Ashtekar H. (2024). Molecular docking and network pharmacology study on active compounds of Cyprus rotundus for the treatment of diabetes mellitus. In Silico Pharmacol..

[50] Xue Z., Cao Z., Jin M., Zhang X., Wang X., Dou J., Zhu Y., Ito Y., Guo Z. (2021). New steroid saponins from Dioscorea zingiberensis yam and their medicinal use against I/R via anti-inflammatory effect. Food Funct..

[51] Zhang X., Wang X., Xue Z., Zhan G., Ito Y., Guo Z. (2021). Prevention properties on cerebral ischemia reperfusion of medicine food homologous Dioscorea yam-derived diosgenin based on mediation of potential targets. Food Chem..

[52] Alharazi W.Z., McGowen A., Rose P., Jethwa P.H. (2022). Could consumption of yam (Dioscorea) or its extract be beneficial in controlling glycaemia: A systematic review. Br. J. Nutr..

[53] Ma J., Meng X., Liu Y., Yin C., Zhang T., Wang P., Park Y.K., Jung H.W. (2020). Effects of a rhizome aqueous extract of Dioscorea batatas and its bioactive compound, allantoin in high fat diet and streptozotocin-induced diabetic mice and the regulation of liver, pancreas and skeletal muscle dysfunction. J. Ethnopharmacol..

[54] Lin Y., Qiu L., Zhang M., Zhang C., Qin Y., Yu H., Lin Q., Ge L. (2025). Comprehensive evaluation on nutritional characteristics and anti-hyperglycemic active ingredients of different varieties of Yam. Sci. Rep..

[55] Hashimoto N., Noda T., Kim S.-J., Sarker M.Z.I., Yamauchi H., Takigawa S., Matsuura-Endo C., Suzuki T., Han K.-H., Fukushima M. (2009). Yam contributes to improvement of glucose metabolism in rats. Plant Foods Hum. Nutr..

[56] Zhang L., Wang S., Zhang W., Chang G., Guo L., Li X., Gao W. (2024). Prospects of yam (Dioscorea) polysaccharides: Structural features, bioactivities and applications. Food Chem..

[57] Feng X., Zhang Q., Li J., Bie N., Li C., Lian R., Qin L., Feng Y., Wang C. (2022). The impact of a novel Chinese yam-derived polysaccharide on blood glucose control in HFD and STZ-induced diabetic C57BL/6 mice. Food Funct..

[58] Guo Y., Liu F., Zhang J., Chen J., Chen W., Hong Y., Hu J., Liu Q. (2024). Research progress on the structure, derivatives, pharmacological activity, and drug carrier capacity of Chinese yam polysaccharides: A review. Int. J. Biol. Macromol..

[59] Li Q., Li W., Gao Q., Zou Y. (2017). Hypoglycemic Effect of Chinese Yam (Dioscorea opposita rhizoma) Polysaccharide in Different Structure and Molecular Weight. J. Food Sci..

[60] Obaroakpo J.U., Liu L., Zhang S., Lu J., Pang X., Lv J. (2019). α-Glucosidase and ACE dual inhibitory protein hydrolysates and peptide fractions of sprouted quinoa yoghurt beverages inoculated with Lactobacillus casei. Food Chem..

[61] Li Y., Fan Y., Liu J., Meng Z., Huang A., Xu F., Wang X. (2023). Identification, characterization and in vitro activity of hypoglycemic peptides in whey hydrolysates from rubing cheese by-product. Food Res. Int..

[62] Abbasi S., Moslehishad M., Salami M. (2022). Antioxidant and alpha-glucosidase enzyme inhibitory properties of hydrolyzed protein and bioactive peptides of quinoa. Int. J. Biol. Macromol..

[63] Taheri R., Mokhtari Y., Yousefi A.M., Bashash D. (2024). The PI3K/Akt signaling axis and type 2 diabetes mellitus (T2DM): From mechanistic insights into possible therapeutic targets. Cell Biol. Int..

[64] Li M., Liu G., Jin X., Guo H., Setrerrahmane S., Xu X., Li T., Lin Y., Xu H. (2022). Micropeptide MIAC inhibits the tumor progression by interacting with AQP2 and inhibiting EREG/EGFR signaling in renal cell carcinoma. Mol. Cancer.

[65] Liu H., Ju A., Dong X., Luo Z., Tang J., Ma B., Fu Y., Luo Y. (2023). Young and undamaged recombinant albumin alleviates T2DM by improving hepatic glycolysis through EGFR and protecting islet β cells in mice. J. Transl. Med..

[66] Shen C., Wang Y., Zhang H., Li W., Chen W., Kuang M., Song Y., Zhong Z. (2023). Exploring the active components and potential mechanisms of Rosa roxburghii Tratt in treating type 2 diabetes mellitus based on UPLC-Q-exactive Orbitrap/MS and network pharmacology. Chin. Med..

[67] Ye C., Li Y., Shi J., He L., Shi X., Yang W., Lei W., Quan S., Lan X., Liu S. (2025). Network pharmacology analysis revealed the mechanism and active compounds of jiao tai wan in the treatment of type 2 diabetes mellitus via SRC/PI3K/AKT signaling. J. Ethnopharmacol..

[68] Xu L., Li Y., Yin L., Qi Y., Sun H., Sun P., Xu M., Tang Z., Peng J. (2018). miR-125a-5p ameliorates hepatic glycolipid metabolism disorder in type 2 diabetes mellitus through targeting of STAT3. Theranostics.

[69] Sun M., Han Z., Luo Z., Ge L., Zhang X., Feng K., Zhang G., Xu F., Zhou H., Han H. (2024). PTPN11 is a potential biomarker for type 2 diabetes mellitus complicated with colorectal cancer. Sci. Rep..

[70] Mao Y., Sun J., Wang Z., Liu Y., Sun J., Wei Z., Wang M., Yang Y. (2023). Combining transcriptomic analysis and network pharmacology to explore the mechanism by which Shaofu Zhuyu decoction improves diabetes mellitus erectile dysfunction. Phytomedicine.

[71] Cui X., Qian D.W., Jiang S., Shang E.X., Zhu Z.H., Duan J.A. (2018). Scutellariae Radix and Coptidis Rhizoma Improve Glucose and Lipid Metabolism in T2DM Rats via Regulation of the Metabolic Profiling and MAPK/PI3K/Akt Signaling Pathway. Int. J. Mol. Sci..

[72] Yao W., Huo J., Ji J., Liu K., Tao P. (2024). Elucidating the role of gut microbiota metabolites in diabetes by employing network pharmacology. Mol. Med..

[73] He C., Wang K., Xia J., Qian D., Guo J., Zhong L., Tang D., Chen X., Peng W., Chen Y. (2023). Natural exosomes-like nanoparticles in mung bean sprouts possesses anti-diabetic effects via activation of PI3K/Akt/GLUT4/GSK-3β signaling pathway. J. Nanobiotechnol..

[74] Ramasubbu K., Devi Rajeswari V. (2023). Impairment of insulin signaling pathway PI3K/Akt/mTOR and insulin resistance induced AGEs on diabetes mellitus and neurodegenerative diseases: A perspective review. Mol. Cell. Biochem..

[75] Eickelschulte S., Hartwig S., Leiser B., Lehr S., Joschko V., Chokkalingam M., Chadt A., Al-Hasani H. (2021). AKT/AMPK-mediated phosphorylation of TBC1D4 disrupts the interaction with insulin-regulated aminopeptidase. J. Biol. Chem..

[76] Zhao H., Zhai B.W., Zhang M.Y., Huang H., Zhu H.L., Yang H., Ni H.Y., Fu Y.J. (2024). Phlorizin from Lithocarpus litseifolius [Hance] Chun ameliorates FFA-induced insulin resistance by regulating AMPK/PI3K/AKT signaling pathway. Phytomedicine.

[77] Chen D., Chen X., He C., Xiao C., Chen Z., Chen Q., Chen J., Bo H. (2023). Sanhuang xiexin decoction synergizes insulin/PI3K-Akt/FoxO signaling pathway to inhibit hepatic glucose production and alleviate T2DM. J. Ethnopharmacol..

[78] Ji P., Shi Q., Liu Y., Han M., Su Y., Sun R., Zhou H., Li W., Li W. (2023). Ginsenoside Rg1 treatment alleviates renal fibrosis by inhibiting the NOX4-MAPK pathway in T2DM mice. Ren. Fail..

[79] Li J.S., Ji T., Su S.L., Zhu Y., Chen X.L., Shang E.X., Guo S., Qian D.W., Duan J.A. (2022). Mulberry leaves ameliorate diabetes via regulating metabolic profiling and AGEs/RAGE and p38 MAPK/NF-κB pathway. J. Ethnopharmacol..

